# Exopolysaccharide from *Lacticaseibacillus paracasei* alleviates gastritis in *Helicobacter pylori*-infected mice by regulating gastric microbiota

**DOI:** 10.3389/fnut.2024.1426358

**Published:** 2024-06-24

**Authors:** Jianxing Yu, Ziqi Chen, Qingqing Zhou, Ping Li, Shiying Wu, Tao Zhou, Qing Gu

**Affiliations:** ^1^Zhejiang Key Laboratory of Food Microbiology and Nutritional Health, College of Food Science and Biotechnology, Zhejiang Gongshang University, Hangzhou, China; ^2^College of Biological, Chemical Sciences and Engineering, Jiaxing University, Jiaxing, China

**Keywords:** *Lacticaseibacillus paracasei*, Exopolysaccharides, *Helicobacter pylori*, Gastritis, Gastric microbiota

## Abstract

**Introduction:**

Many probiotics have the ability to produce extracellular polysaccharides (EPS). EPS derived from these probiotics has been confirmed to regulate the host intestinal microecological balance and alleviate the symptoms of diseases caused by gastrointestinal microecological imbalance.

**Results:**

Lactic acid bacteria (LAB) strain with good exopolysaccharide (EPS) producing ability, namely, *Lacticaseibacillus paracasei* ZFM54 (*L. paracasei* ZFM54) was screened. The fermentation conditions of *L. paracasei* ZFM54 for EPS production were optimized. The EPS54 was characterized by chemical component and monosaccharide composition determination, UV, FT-IR and NMR spectra analysis. Cango red, SEM, AFM and XRD analysis were conducted to characterize the structure of EPS54. The EPS54 effectively reduced the colonization of *Helicobacter pylori* to AGS cells and recovered the cell morphology. EPS54 could also effectively alleviate the gastritis in the *H. pylori*-infected mice by down-regulating the mRNA expression levels of pro-inflammatory cytokines IL-6, IL-8, IL-1β and TNF-α and up-regulating the mRNA expression of inflammatory cytokine IL-10 in gastric cells. EPS54 was also found to be able to positively regulate the structure of gastric microbiota.

**Conclusion:**

The EPS 54 from *L. paracasei* ZFM54 can alleviate gastritis in *H. pylori*-infected mice by modulating the gastric microbiota.

## 1 Introduction

Lactic acid bacteria (LAB) is the general name of a group of bacteria that can ferment sugars to produce lactic acid. They are distributed in wild environments and living things from soil to mammalian oral cavity and gastrointestinal tract. Some Gram-positive strains of LAB are considered as beneficial bacteria to human health, and the common bacteria in human intestinal tract, thus are generally recognized as safe (GRAS) edible bacteria (1, 2). It is generally accepted that there is a direct relationship between functions of the probiotics and their secondary metabolites. Therefore, functions, action mechanism and application of LABs and their metabolites have become research hotspots lately. Functional bacteria have been demonstrated to improve the intestinal microenvironment (3, 4), and exert anti-inflammatory (5, 6), immune-stimulatory (7, 8), and anti-tumoral effects (9) mainly by colonizing in the intestine, and secreting multiple-functional compounds, such as antibacterial peptides (10), polysaccharides (11–13), short-chain fatty acids (14, 15), and vitamins (16, 17). Among them, polysaccharides, as the secondary metabolites of the bacteria, are identified as key bioactive molecules in probiotic characteristics, however the beneficial effect of EPS production on the bacterial physiology has still not been fully clarified (18, 19).

Many LABs in nature have the ability to produce EPSs, most of which were found in milk and dairy products, traditional fermented foods and animal intestines. In recent years, more attention has been paid to the properties and physiological functions of EPSs from LABs as the demand of consumers turns to natural, residue free, safe and healthy food. Therefore, it is of great significance to search for LAB strains with good EPS-producing ability and study the biological activities and action mechanisms of EPSs. EPSs from other probiotics have been demonstrated to regulate the balance of intestinal microecology of the host and alleviate symptoms of the diseases caused by the imbalance of intestinal microecology (20, 21). It was reported that the EPS from *Bifidobacterium breve* UCC2003 significantly improved the colonization ability of the strain and play its immunomodulatory role (22). Thus, EPSs from probiotics can be applied in yogurt, cheese milk and other foods as prebiotics to improve the food quality (23). Although probiotics have been revealed to be able to ameliorate imbalance of gastric microbiota and gastritis induced by *Helicobacter pylori* (23), such effects of EPSs from probiotics have rarely been reported. Herein, the LAB strains stored in our laboratory were screened for their EPS-producing capacity, the effects of EPS produced by *L. paracasei* ZFM54 (CCTCC NO:2016667) on the inflammation and microbiota composition in the stomach were also investigated using a *Helicobacter pylori*-infected mouse model.

## 2 Materials and methods

### 2.1 Materials

The LAB strains used for the screening of EPS-producing ability were preserved in our lab, which were isolated from milk, pickled vegetables, and infant feces. *Helicobacter pylori* ZJC03 (CCTCC NO: M 20211218), kindly provided by a hospital, was isolated from the gastric mucosa of patients with gastritis, and cultured on *H. pylori* medium (Hopebio, Qingdao, China) broth supplemented with 7% sheep blood (HPM-S) under microaerobic conditions at 37°C using a gas generating kit (Mitsubishi GasChemical, Tokyo, Japan).

RNA Extraction Kit was the product of Servicebio (Wuhan, China), the PrimeScript RT Reagent Kit with gDNA Eraser was the product of Takara (Japan), the NucleoSpinSoil Kit was purchased from Macherey-Nagel (Düren, Germany). The species-specific primers were synthesized by Invitrogen (China).

### 2.2 Screening of EPS producing LABs

LABs were activated on MRS agar plates twice, and cultured at 37°C for 72 h. Possible EPS-producing strains were identified via their mucoid or ropy appearance (24, 25). The potential EPS-producing strains were inoculated into 100 mL MRS broth media at a dose of 2% (v/v). After 48 h cultivation, trichloroacetic acid (TCA) was added to the broth to reach a final concentration of 8% (w/v), followed by centrifugation (10,000 × g, 20 min) to remove the bacterial cells and proteins (26). The supernatant was concentrated to one fourth of the original volume, followed by the addition of 3 folds volume of ethanol. The yielding mixture was allowed to stand in a fridge overnight. The yielding precipitate obtained by centrifugation (10,000 × g, 20 min) was redissolved in deionized water, followed by dialysis (Mw cut-off 3,500 Da) for 72 h. After lyophilization, the EPS was obtained as a powder. Phenol-sulfuric acid method was applied to determine the total sugar content of EPS using glucose as a standard (27). The strain possessing the highest EPS production was selected for further investigation.

### 2.3 Optimization of EPS fermentation conditions for EPS production

#### 2.3.1 Effect of carbon source on EPS production

MRS broth without glucose was applied as an initial medium for the optimization of carbon source. After addition of sugar (glucose, galactose, fructose, lactose and sucrose) to MRS broth (final concentration of 2%, w/v), the yielding broth was adjusted to pH 6.8. The strain was inoculated at a dose of 2% (v/v), followed by fermentation under static conditions at 37°C for 24 h. The EPS was isolated from the broth as described in section 2.2.

#### 2.3.2 Effect of nitrogen source on EPS production

Peptone, tryptone, casein peptone, yeast extract and ammonium nitrate (final concentration of 0.5%, w/v) were added to the original medium, and the pH of the fermentation broth was adjusted to 6.8. The strain was inoculated at a dose of 2% (v/v), followed by fermentation under static conditions at 37°C for 24 h. The EPS was isolated from the broth as described in section 2.2.

#### 2.3.3 Effect of initial pH on EPS production

The pH was adjusted to 4, 5, 6, 7 and 8, and the strains were inoculated at a dose of 2% (v/v), and then fermented at 37°C for 24 h to determine the yield of exopolysaccharides according to the section 2.2.

#### 2.3.4 Effect of inoculation dosage on EPS production

EPS production obtained at different inoculum dosage of strain ranging from 0.5 to 4% (v/v) in MRS broth were examined. Fermentation was performed at 37°C for 24 h. EPS isolation and determination were performed as described in section 2.2.

#### 2.3.5 Effect of fermentation time on EPS production

The strain was inoculated at a dose of 2% (v/v) in MRS broth, and then fermented under static conditions at 37°C, and the yield of EPS was determined according to section 2.2 at every 12 h during the fermentation time of 12–48 h.

#### 2.3.6 Effect of fermentation temperature on EPS production

Effect of fermentation temperature on EPS production was investigated by fermenting the specific bacterial strain in MRS broth at a dose of 2% (v/v) at 27°C, 32°C, 37°C and 42°C. EPS production was determined as described in section 2.2.

### 2.4 Characterization of exopolysaccharide

#### 2.4.1 Chemical composition assay

The total sugar content of EPS was determined by the phenol sulfuric acid method using mannose as a standard (27), sulfate content was determined using barium sulfate turbidimetric method (28), uronic acid was determined by hydroxy biphenyl method, and protein was analyzed by Coomassie brilliant blue method (29).

#### 2.4.2 Monosaccharide composition measurement

Monosaccharide composition of the EPS was measured using a 1-phenyl-3-methyl-5-pyrazolone (PMP) derivatization method by HPLC (30). Briefly, EPS sample (20 mg) were hydrolyzed with trifluoroacetic acid (TFA, 5 mL, 2 M) in a 20 mL sealed clamp bottle under nitrogen atmosphere. The hydrolysis was performed in an oven at 100°C for 2 h. After cooling, the hydrolyzed solution (1 mL) was transferred to a test tube and mixed with methanol (1 mL), then evaporated with N_2_ for 3 times at 70°C to remove TFA completely. The residue was dissolved in NaOH solution (1 mL, 0.3 M) to obtain the EPS hydrolysate. The hydrolysate was derivatized with PMP (400 μL) at 70°C for 2 h. After cooling to room temperature, the resulting solution was neutralized to pH 6–7 with 0.3 M HCl, then 1,200 μL of double distilled H_2_O was added, followed by the addition of the same volume of chloroform for extracting PMP residue. The aqueous layer was extracted with chloroform twice to completely remove the PMP residue. Finally, the aqueous phase was filtered by 0.45 μm microporous membrane prior to HPLC analysis. The monosaccharide standards (glucose, galactosamine, galactose, xylose, arabinose, L-fucose, guluronic acid, mannose uronic acid, mannose, ribose, rhamnose, glucosamine, glucuronic acid, galacturonic acid) were derivatized with PMP using the same method. The HPLC analysis was performed on an Agilent 1100 system (Agilent, United States) equipped with an C_18_ column (250 mm × 4.6 mm i.d., 5 μm particle size, Waters, United States), with injection volume of 5 μL and detection wavelength of 250 nm. The mobile phase consisted of 100 mM disodium hydrogen phosphate and acetonitrile with a gradient elution program (1 mL/min, 30°C).

#### 2.4.3 Molecular weight distribution measurement

The Molecular weight (Mw) distributions of the EPS (50 mg/mL, injection volume: 50 μL) was analyzed using HPSEC with refractive index (RI) detection. Analysis was performed on an Agilent 1,100 system (Agilent, United States) equipped with an Ultrahydrogel Linear column (300 mm × 7.8 mm i.d., 10 μm particle size, Waters, United States). Disodium hydrogen phosphate (60 mM) was used as an eluent at a flow rate of 0.5 mL/min at a column temperature of 30°C. Dextran standards (670 kDa, 150 kDa, 250 kDa, 5 kDa, Sigma Aldrich, Denmark) were used for the calibration of the molecular weight of the EPS.

#### 2.4.4 UV, FT-IR and NMR spectra analysis

UV spectrum of EPS (1 mg/mL) was obtained by recording on an ultraviolet–visible spectrophotometer (UV-2550, Shimadzu, Tokyo, Japan) in the range of 200–800 nm.

FT-IR spectrum of EPS sample was recorded on a Nicolet 380 infrared spectrometer (Thermo Fisher, United States) using a KBr pellet method scanning in the range of 400 to 4,000 cm^−1^ (31).

^1^H NMR and ^13^C NMR spectra of EPS (60 mg in 600 μL D_2_O) were determined on a Bruker Avance 500 NMR instrument (31).

#### 2.4.5 Congo red test

The sample solution (0.1 mg/mL) and Congo red solution (80 μmol/L) were mixed evenly in the test tube (ultrapure water was used as a negative control), and the amount of NaOH solution (1 mol/L) was adjusted to make the final concentration of sodium hydroxide in the reaction system to be 0, 0.01, 0.05, 0.1, 0.2, 0.3, 0.4, 0.5 mol/L, respectively. After homogeneous mixing, the samples were scanned with a UV spectrophotometer in the range of 400 to 600 nm, and the maximum absorption wavelength was recorded (31).

#### 2.4.6 SEM, AFM and XRD analysis

The dry sample of EPS54 was taken and dispersed on the surface of the conductive adhesive, and then the sample that was not adhered to the conductive adhesive was gently blown off, so that the EPS54 could form a uniform thin layer on the conductive adhesive. Subsequently, the gold-sprayed samples were placed on the Phenom ProX Desktop SEM to observe the microscopic appearance of EPS54 and the images were recorded (31).

The EPS54 solution was first shaken by ultrasound for 5 min to reduce sample clumping, and then thoroughly stirred for 2 h in a water bath at 50°C to fully disperse. After cooling to room temperature, the sample solution was filtered through a 0.45 μm microporous filter membrane and then used for testing. AFM determination of EPS54 was performed with a Dimension Icon atomic force microscope and images were recorded (31).

XRD tests were performed with a Bruker D8ADVANCE X-ray diffractometer. The diffraction conditions were set as follows: copper target (*k* = 1.5406 A), tube pressure 40 kV, Angle range 3–80° (2θ), scanning speed 0.5°/s (31).

### 2.5 Effect of EPS on *Helicobacter pylori* infected AGS cells

Sterile cell slides were placed in 6-well plates, and 1 mL AGS cells of 1 × 10^6^ cells/mL were added to each well. After cell adherence, 1 mL of 1 × 10^8^ CFU/mL *H. pylori* ZJC03 resuspended in Ham’s F-12K without double antibody medium was added and incubated with AGS cells for 2 h in a cell incubator (37°C, 5% CO_2_). Nonadherent *H. pylori* ZJC03 was removed by gentle rinsing several times with PBS. The cell slides were left in 4% paraformaldehyde for 15 min at room temperature, and then the surface liquid was carefully blotted dry. Then the cell slides were stained with oxalate crystal violet, rinsing, iodine solution staining, ethanol decalcification, and safranin counterstained for Gram staining.

Urease activity was determined by the phenol red method. A 96-well plate was taken and 100 μL of 2 × 10^5^ cells/mL AGS cells in the logarithmic growth phase was added to each well. After the cells adhered to the wall, the culture medium was removed and the following procedures were performed: (1) 100 μL resuspension of 1 × 10^6^ CFU/mL *H. pylori* ZJC03 resuspended in Ham’s F-12K without double antibody medium was added as control group. (2) 100 μL resuspension of 1 × 10^6^ CFU/mL *H. pylori* ZJC03 resuspended in Ham’s F-12′K nonantibody medium (containing EPS54 600 μg/mL) was added as test group. (3) 100 μL Ham’s F-12K complete medium was added as blank control group. Each group consisted of three parallel sets. AGS cells were incubated for 2 h in a cell incubator (37°C, 5% CO_2_). After incubation, phenol red reagent was added and the OD_550_ of each well was determined.

### 2.6 Effect of EPS on *Helicobacter pylori* infected mice

#### 2.6.1 Animal test design

The animal experiments were conducted in accordance with the Guidelines for Care and Use of Laboratory Animals and approved by the Animal Ethics Committee of Shanghai Public Health Clinical Center (Shanghai, China) with the protocol number 2021-A044-01. Female specific pathogen-free (SPF) C57BL/6J mice (6 weeks old, 16–18 g) were provided by Sino British SIPPR/BK Lab Animal Ltd. (Shanghai, China). Adaptive culture and feeding of the mice were performed in the Laboratory Animal Department of the Shanghai Public Health Clinical Center. The experimental design for evaluating the effects of EPS54 on *H. pylori-*infected mice was depicted in Supplementary Figure S1.

After 1 week of adaptive culture, the mice were randomly assigned to seven groups (10 mice each group): Control, HP, E5, E10, E20, T, and E_T groups. The details of these groups are provided below:

Group Control: the mice were orally administered with 400 μL of sterile HPM-S broth once every 2 days for two consecutive weeks, followed by oral gavage with 400 μL of sterile water every day for four consecutive weeks. Groups HP, E5, E10, E20, T, E_T: the mice were orally administered with 400 μL of *H. pylori* (10^8^ CFU/mL) at two-day intervals for two consecutive weeks to achieve infection. Then, the mice in groups HP, E5, E10, E20, T and E_T were orally administered with 400 μL of corresponding testing solutions (HP: sterile water; E5: 5 g/L EPS54; E10:10 g/L EPS 54; E20: 20 g/L EPS54; T: triple therapy (82.5 mg/L omeprazole +2.1 g/L amoxicillin +12.5 g/L clarithromycin) for the first 10 days, and sterile water for the following 18 days; E_T: EPS + triple therapy (10 g/L EPS54 + 41.25 mg/L omeprazole + 1.05 g/L amoxicillin + 6.25 g/L clarithromycin) for the first 10 days, and 10 g/L EPS54 for the following 18 days) every day for four consecutive weeks.

In all the groups, the mice were sacrificed by cervical dislocation after treatment. Gastric mucosal samples were cleaned with a sterile saline solution and collected aseptically. Gastric contents were frozen in liquid nitrogen and kept at −80°C until analysis.

#### 2.6.2 Histopathological observation of gastric tissue

The gastric mucosa was pretreated and observed to illustrate the damage caused by *H. pylori* according to a previous study (32). Briefly, the mucosa was fixed in 4% paraformaldehyde buffer solution (pH 7.4) for 36 h. The tissues were then embedded in paraffin, sectioned at 5 mm, and stained with hematoxylin and eosin. The stained slides were observed under an optical microscope (Nikon Eclipse Ci, Japan); representative images (200 × magnification) were randomly selected.

#### 2.6.3 Real-time quantitative polymerase chain reaction assay

Real-time quantitative polymerase chain reaction (RT-qPCR) was used for the quantitative analysis of inflammatory factors in the gastric cells, including IL-6, IL-1β, IL-8, IL-10 and TNF-α. Total RNA was isolated from the gastric mucosa of the mice by the RNA Extraction Kit. Then RNA (500 mg) was reverse-transcribed into cDNA using the RT reagent kit. Glyceraldehyde-3phosphate dehydrogenase (GAPDH) was used as a control. The RT-qPCR assay was performed using SYBR Premix Ex Taq II as per the following thermocycling program: a preamplification stage at 95°C for 10 min, followed by 40 cycles at 95°C for 15 s and 60°C for 1 min. All mRNA quantitative data were normalized to GADPH values and were calculated by the 2^−ΔΔCt^ method (33). The data are presented as fold changes over the control using the critical threshold (Ct). The primer sequences are as follows in Table 1.

**Table 1:** Primer sequences for quantitative real-time polymerase chain reaction.

Gene	Primer sequences
GAPDH	F: CCTCGTCCCGTAGACAAAATG
	R: TGAGGTCAATGAAGGGGTCGT
IL-1β	F: TGCCACCTTTTGACAGTGATG
	R: CATCTCGGAGCCTGTAGTGC
IL-6	F: TTCTTGGGACTGATGCTGGTG
	R: CACAACTCTTTTCTCATTTCCACGA
TNF-α	F: TGGAACTGGCAGAAGAGGCAC
	R: AGGGTCTGGGCCATAGAACTGA
IL-10	F: TTACCTGGTAGAAGTGATGCCC
	R: GACACCTTGGTCTTGGAGCTTA
IL-8	F: TAGGCATCTTCGTCCGTCCC
	R: GCCAACAGTAGCCTTCACCCA

#### 2.6.4 Microbiota analysis by 16S rRNA gene sequencing

DNA samples from the gastric contents were extracted by the NucleoSpinSoil Kit. Then, V3-V4 regions of bacterial 16S rRNA genes were amplified and sequenced on the Illumina MiSeq platform at LC-Bio Technology Co., Ltd. (Hangzhou, China). Raw data were synthesized by sequencing the libraries on the NovaSeq PE250 platform and were then analyzed on the cloud platform^1^ to evaluate the diversity of gastric microbiota.

### 2.7 Statistical analysis

All data are represented as the mean ± standard deviation (SD). The data were analyzed by ordinary one-way ANOVA with tukey and LSD test using GraphPad Prism version 9.0 software (GraphPad Software Inc., San Diego, CA, United States). The error bar represents SD based on the average values of at least three replicates. The differences between the treatments were considered statistically significant at ^*^p < 0.05 and ^**^p < 0.01.

## 3 Results

### 3.1 Screening of EPS producing strains

The colonies of EPS-producing lactic acid bacteria were moist and viscous, and there were obvious transparent circles around the colonies. Fifty strains isolated from fresh milk and stool samples of healthy infants were used for initial screening. Among these strains, fourteen strains were preliminary screened for their EPS producing capability, including *Enterococcus faecium*, *Enterococcus durans*, *Lacticaseibacillus rhamnosus*, *Lactiplantibacillus plantarum*, *Latilactobacillus sakei*, *Lacticaseibacillus paracasei* (Table 2). The EPS productions varied from 99.8 μg/mL to 307.3 μg/mL. *Lacticaseibacillus paracasei* ZFM54 was found to possess the best ability to produce EPS among these LAB strains, thus it was selected for EPS preparation. In addition, *L. paracasei* ZFM54 has been identified in our previous work (32, 34, 35).

**Table 2:** LAB strains and their EPS producing capacities.

Number	Strains	EPS yield (mg/L)
1	*Enterococcus faecium* ZFM215	99.8
2	*Lacticaseibacillus rhamnosus* ZFM202	131.0
3	*Lactiplantibacillus plantarum* ZFM9	134.6
4	*Lactiplantibacillus plantarum* ZFM228	143.2
5	*Latilactobacillus sakei* ZFM223	158.0
6	*Latilactobacillus sakei* ZFM221	177.7
7	*Enterococcus faecium* ZFM45	187.8
8	*Lacticaseibacillus rhamnosus* ZFM216	216.6
9	*Lactiplantibacillus plantarum* ZFM4	285.1
10	*Enterococcus durans* ZFM92	285.8
11	*Lactiplantibacillus plantarum* ZJ316	288.8
12	*Lactiplantibacillus plantarum* ZFM206	290.0
13	*Lactiplantibacillus plantarum* ZFM55	293.7
**14**	***Lacticaseibacillus paracasei* ZFM54**	**307.3**

### 3.2 Optimization of fermentation conditions of *Lacticaseibacillus paracasei* ZFM54

#### 3.2.1 Influence of carbon source on EPS production

**Figure 1:**
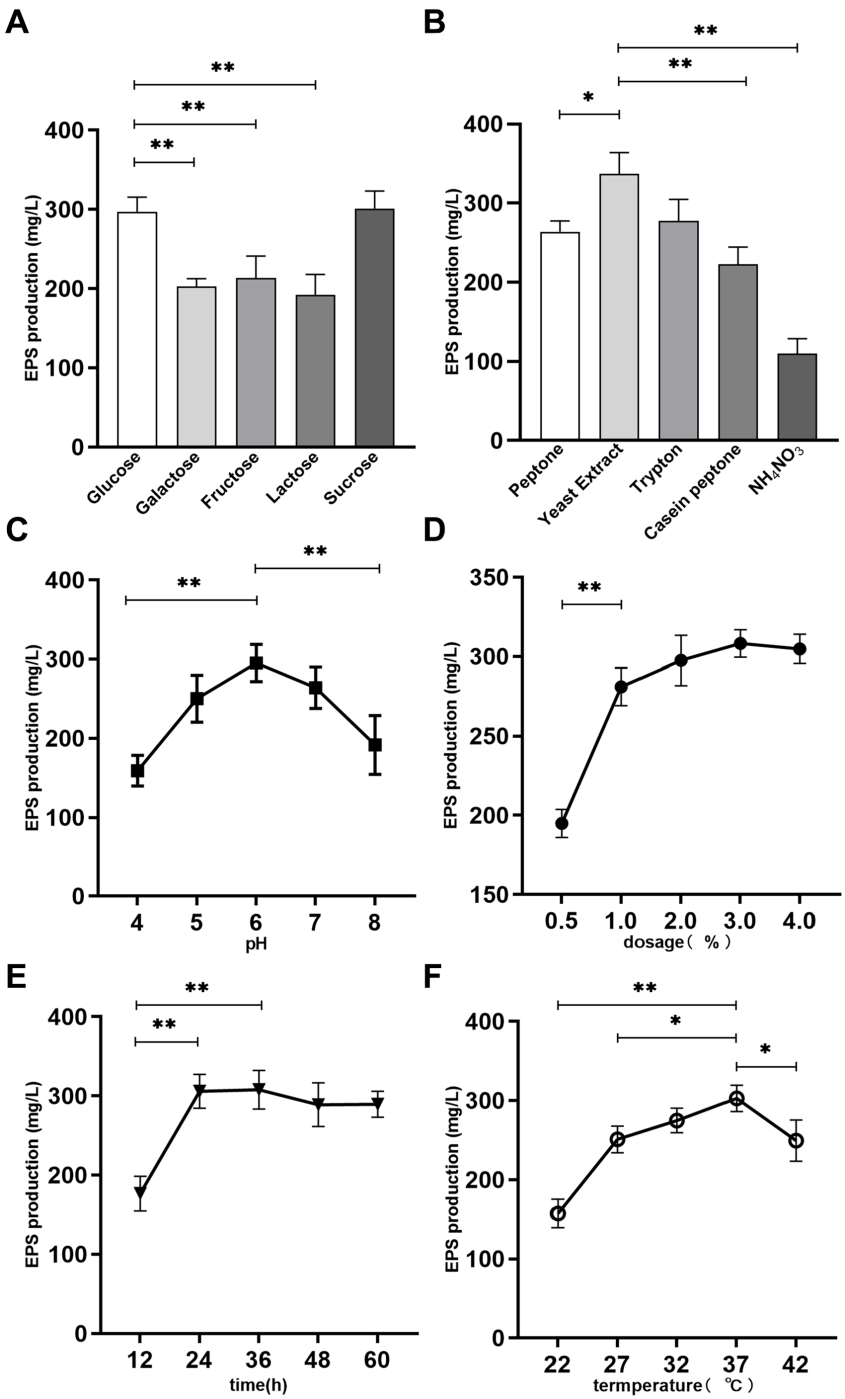
Effects of medium composition and fermentation conditions on EPS production. **(A)** Carbon source. **(B)** Nitrogen source. **(C)** pH. **(D)** Temperature. **(E)** Fermentation time. **(F)** Dosage. ^*^p < 0.05 and ^**^p < 0.01.

*Lacticaseibacillus paracasei* ZFM54 was fermented in the MRS broth (glucose free) using 5 kinds of sugars as carbon source under same culture conditions, so as to assess the effect of carbon source on EPS production. There was a statistically significant (*p* < 0.05) difference in EPS production among the different carbon sources after fermentation. As presented in Figure 1A, *L. paracasei* ZFM54 cultured in sucrose MRS possessed the best EPS producing ability, reaching 301.2 ± 18.1 mg/L, followed by that in glucose MRS (297.1 ± 15.2 mg/L); while the EPS production in lactose MRS was the lowest (192.3 ± 20.8 mg/L). The EPS yield using the five carbon sources followed the order: sucrose≈glucose>fructose>galactose>lactose. In a previous report (36), sucrose was also found to be the best carbon source for the EPS production by *Streptococcus thermophilus* 05-34.

The EPS production by *L. paracasei* ZFM54 using sucrose and glucose carbon source was significantly higher than that using other sugars (*p* < 0.01). Considering that the glucose is the recommended carbon source in MRS medium components, glucose was chosen as the carbon source for *L. paracasei* ZFM54 fermentation in this study.

#### 3.2.2 Influence of nitrogen source on EPS production

Sufficient and available nitrogen sources in the medium can promote microbial growth and the biosynthesis of secondary metabolites such as polysaccharides. In the present work, four organic nitrogen sources and one inorganic nitrogen source were used to examine their effects on EPS production. As presented in Figure 1B, the yield of EPS varied significantly in the presence of different nitrogen sources (*p* < 0.05). Among them, the EPS yield corresponding to yeast extract was the highest, reaching 336.8 ± 7.1 mg/L, tryptone (277.6 ± 27.3 mg/L) and peptone (263.9 ± 13.5 mg/L) the next, and casein peptone the lowest (222.8 ± 21.8 mg/L). Ammonium nitrate was less effective than the above four organic nitrogen sources, and EPS production was only 109.8 ± 19.1 mg/L. This is possibly because inorganic nitrogen source needs to be transformed to organic nitrogen source through the action of a variety of metabolic enzymes prior to the utilization by the bacteria, which affects the synthesis efficiency of protein and other materials, thus delaying the growth and sugar production of bacteria (37).

#### 3.2.3 Influence of pH on EPS production

The appropriate pH for the growth of LABs generally ranged 4–8, thus the effect of pH on EPS production by *L. paracasei* ZFM54 was investigated in this pH range. As can be seen from Figure 1C, pH 6 was optimal for the EPS production, and a maximum EPS production was obtained at this pH, being 295.1 ± 6.23 mg/L.

#### 3.2.4 Influence of inoculation dosage on EPS production

Inoculation dosage determines the initial cell concentration of the zymotic fluid, thus affecting the EPS production progress. Low inoculation dosage prolongs the incubation period, leading to a reduction in the EPS productivity. As illustrated in Figure 1D, EPS yield increased significantly with the increase of inoculation dosage up to 2% (*p* < 0.01), thereafter the EPS yield did not change obviously. The maximum yield was obtained at 3% inoculation dosage, being 308.3 ± 8.7 mg/L. High inoculation dosage may accelerate the fermentation progress, and shorten the fermentation time. Thus, 1–3% inoculation dosage could be the reasonable dosage.

#### 3.2.5 Influence of fermentation time on EPS production

EPSs belong to microbial secondary metabolites, which are mainly produced during the logarithmic growth and stationary phases of microorganisms. Figure 1E showed the effect of different fermentation times on the yield of EPS. The highest EPS production was observed at 36 h, being 307.8 ± 24.3 mg/L at 36 h, which was very close to that at 24 h (305.0 ± 21.3 mg/L, *p* > 0.05), and was significantly higher than that at 12 h (*p* < 0.01).

#### 3.2.6 Influence of fermentation temperature on EPS production

Temperature affects the activities of enzymes involved in EPS biosynthesis, thereby affecting the production of EPS. Five temperatures commonly used for the bacterial fermentation were selected to examine the influence of fermentation temperature on EPS production (Figure 1F). It was found that the highest EPS yield was obtain at 37°C fermentation, reaching 302.8 ± 16.5 mg/L. When cultured at low temperature, the growth of the strain was suppressed, leading to a low EPS production. In contrast, when growing above the optimum temperature, growth of the strain was activated, and more sugar was consumed to resist the high ambient temperature, thus less energy was used to the produce EPSs, resulting in a low EPS yield (38).

According to the above results, the fermentation conditions of *L. paracasei* ZFM54 for EPS production were ascertained as: glucose as carbon source, yeast extract as nitrogen source, 37°C, 2% inoculation dosage, 24 h. EPS prepared under these fermentation conditions was used in the following investigation.

### 3.3 Characterization of EPS54

#### 3.3.1 Chemical composition, monosaccharide composition, and molecular weight

The total sugar, protein, sulfate, and uronic acid contents of EPS54 were measured as 66.52 ± 0.90%, 2.23 ± 0.46%, 13.53 ± 0.85%, and 11.16 ± 0.85%, respectively. The results showed that the total sugar content in the EPS54 was high, and the protein content was low, indicating that TCA could effectively remove the protein in the fermentation broth during the deprotein process. Both sulfate and uronic acid contents in EPS54 exceeded 11%, which was relatively high. The presence of sulfate and uronic acid indicates that EPS54 is an anion polysaccharide.

Monosaccharide composition assay indicated EPS54 was a heteropolysaccharide, being composed of mannose, galactose, glucose, ribose, glucuronic acid, L-fucose, galactosamine, rhamnose (Supplementary Figure S2), with molar percentage of 68.9, 10.0, 7.7, 7.4, 2.4, 1.5, 1.3, 0.7%, respectively. Mannose residue was found to be the most predominant composition in EPS54. The high proportion of mannose could be because cell wall of yeast, the main raw material of MRS medium, contained a large amount of mannose. Polysaccharides containing L-fucose possess many important physiological functions such as immune regulation, anti-infection and anti-tumor (39). Zhang et al. (40) also found the existence of fucose residues in the extracellular polysaccharide of *L. paracasei*, and speculated that *L. paracasei* had the specific ability to produce fucose.

Molecular weight is an important factor determining its functional characteristics. Previous studies have revealed that the molecular weight of EPS from LABs is generally in a range of 4.0 × 10^4^–6.0 × l0^6^ Da (41, 42). As presented in Supplementary Figure S3B, EPS54 showed a single peak on HPGPC with a retention time of 15.644 min. The Mw of EPS54 was estimated as 170 kDa according to the standard equation (Supplementary Figure S3A).

#### 3.3.2 UV, FT-IR, ^1^H NMR and ^13^C NMR spectra analysis

**Figure 2:**
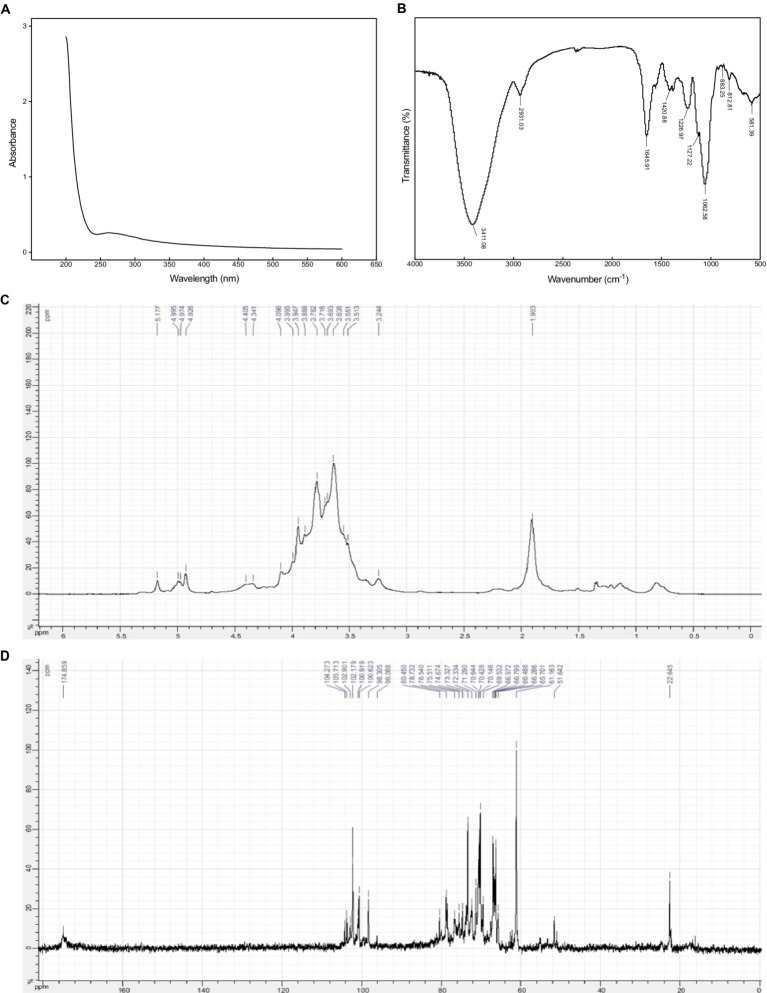
UV **(A)**, FT-IR **(B)**, ^1^H NMR **(C)** and ^13^C NMR **(D)** spectra of EPS54.

As presented in Figure 2A, there was a weak absorption at 280 nm in the UV–Vis spectrum of EPS54, indicating that EPS54 contained low content of protein, which was consistent with the result of chemical component analysis. In addition, no obvious absorption peaks were observed at 260 nm and 380–700 nm, indicating that the absence of nucleic acid and pigment in EPS54, and high purity of the sample.

In the FT-IR spectrum of EPS54 (Figure 2B), the strong wide band at 3411 cm^−1^ is assigned to the stretching vibration of O–H (43); the peak at 2931 cm^−1^ is due to the stretching vibration of saturated C–H (44); the peaks appearing at 1,645 and 1,420 cm^−1^ are attributed to the asymmetric and symmetric stretching of carboxyl group (–COO^−^) in uronic acids. The peak at 1,226 cm^−1^ is assigned to the stretching vibration of O=S=O, indicating the presence of sulfate group, which agrees with the result of chemical composition analysis. The peaks at 1,127 and 1,062 cm^−1^ are the characteristics of C–O vibration (C–O–H, C–O–C) in the pyranose ring. The signals at 812 and 883 cm^−1^ are due to α- and β-type glycosidic bonds (41, 42).

NMR spectra can provide important structural information of polysaccharides. In ^1^H NMR spectra, the anomeric protons in α-pyranoses appear at chemical shifts δ 4.9–5.5 ppm, while those in β-pyranoses are at *δ* 4.3–4.9 ppm (41, 42). In ^13^C NMR spectra, the anomeric carbons in α- and β-pyranoses generally appear at the chemical shifts *δ* 90–102 and 103–110 ppm, respectively. As illustrated in Figure 2C, EPS54 contain both α- and β-glycosidic bonds. This result agrees with that of FT-IR spectra analysis. The signals appearing at δ 98–104 ppm in ^13^C NMR spectrum of EPS54 (Figure 2D) further confirms the presence of both α and β-glycosidic configurations in EPS54. The peak at *δ* 174.8 ppm is assigned to the carbons of carboxyl groups in glucuronic acid residues.

#### 3.3.3 Triple helical structure analysis

**Figure 3:**
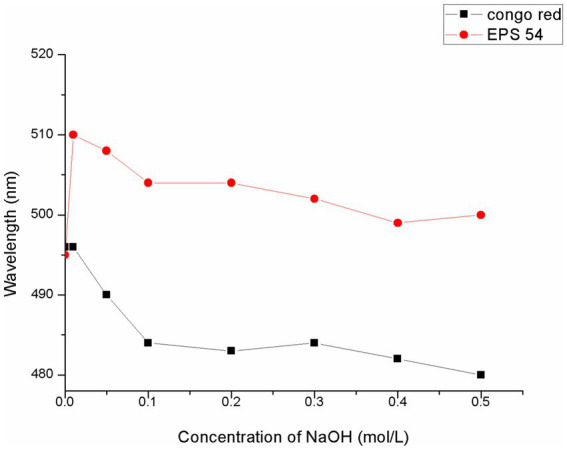
Analysis of spiral curl changes of EPS54 at different NaOH concentrations.

Congo red is an acidic dye that complexes with polysaccharides with a triple-helix conformation. In a certain range of NaOH concentration, the maximum absorption wavelength of the complex was red-shifted compared with that of Congo red solution (the phenomenon of increase in *λ*_max_), which showed that the color of the solution changed to purplish red. When the *λ*_max_ of the polysaccharide-Congo red complex reached above 505 nm in 0.1 mol/L sodium hydroxide solution, it could be considered that the polysaccharide-Congo red complex had a triple helical structure in aqueous solution (45, 46). As presented in Figure 3, the *λ*_max_ of the mixed solution of EPS54 and Congo red was larger than that of the control solution of Congo red at each NaOH concentration, showing an obvious red shift phenomenon. The mixed solution of EPS54 and Congo red in 0.01 M and 0.1 M NaOH had a *λ*_max_ of 510 nm and 505 nm, respectively, and further increase of NaOH concentration resulted in a decrease of *λ*_max_, indicating that EPS54 had a triple-helical conformation.

#### 3.3.4 SEM, AFM and XRD analysis

**Figure 4:**
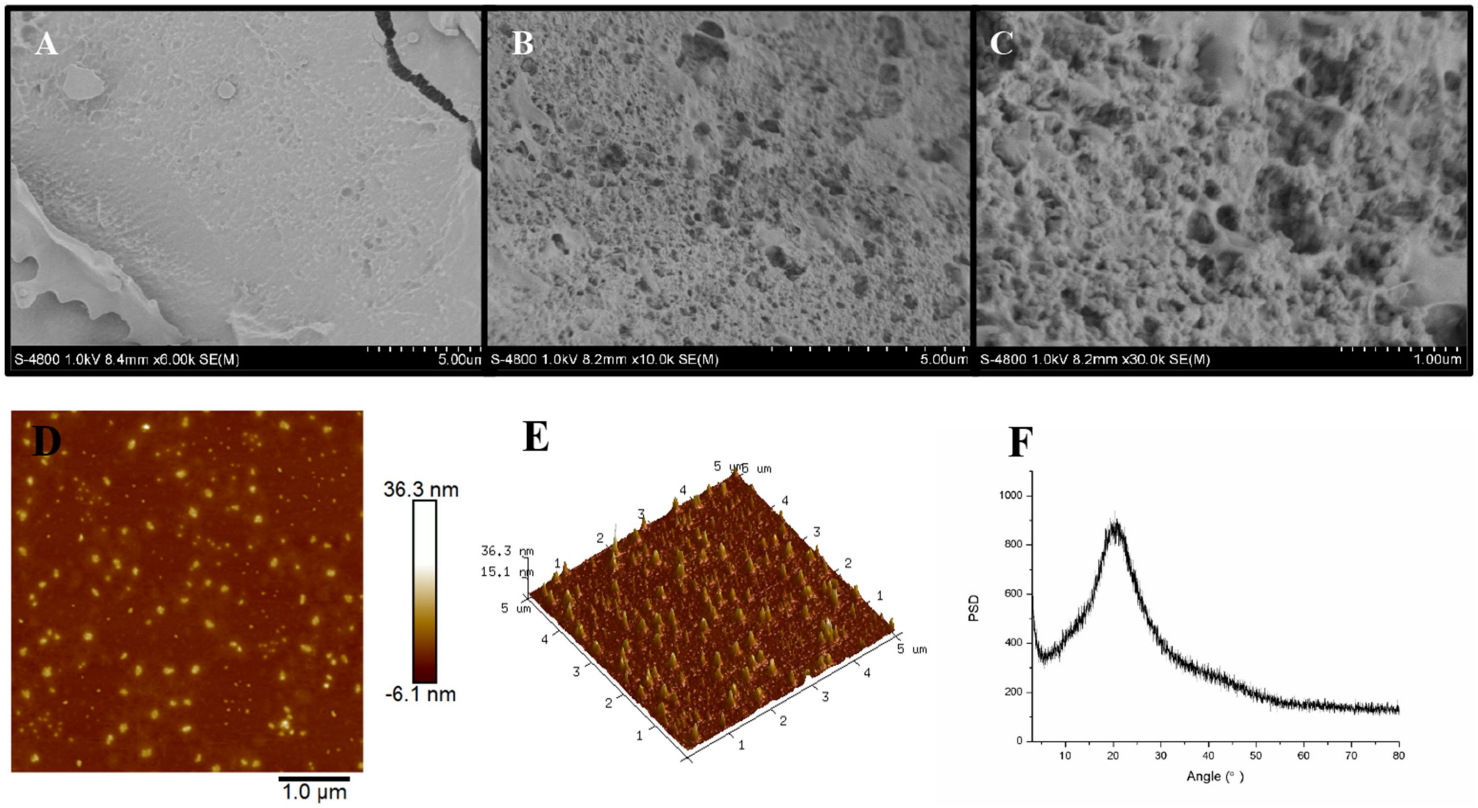
SEM **(A–C)**, AFM **(D,E)** and XRD **(F)** analysis of EPS54.

As shown in SEM image of EPS54 with a 6,000× magnification (Figure 4A), the surface of EPS54 sample is uneven and rough, and there are round cracks and pore-like structures on its surface, which is presumed to be formed due to the formation of ice from the surface moisture and subsequent sublimation during the freeze-drying process. In the SEM images with higher magnification [Figure 4B (10,000×) and Figure 4C (30,000×)], it is found that EPS54 presents an irregular structure, with a rough surface filled with small and large pores, which is similar to the three-dimensional spider network structure of the exopolysaccharide produced by *Rhodococcus erythropolis* HX-2 (47). Such highly complex tangled porous structure was also observed in EPS2, a purified exopolysaccharide from *L. rhamnosus* ZFM216 (30). The highly tangled porous structure of the polysaccharide is generally related to the texture, rheological and biological properties of the polysaccharides (30).

AFM can be used to study the surface structure of macromolecular polymers such as polysaccharides at nanoscale, and analyze their unfolding conformation after solvent dispersion. As illustrated in Figure 4D, EPS54 appears as a spherical shape without completely unfolding to form a chain. The height displayed in the 3D-AFM image is 15.1–36.3 nm, indicating the spherical shape of EPS54 in solution with a molecular size between 15.1–36.3 nm (Figure 4E).

XRD has been widely used in the determination of crystal forms and crystal structures of biological macromolecules such as polysaccharides. In the XRD pattern of EPS54 (Figure 4F), an obvious broad dispersion peak near the diffraction Angle (2θ) 20° was observed, indicating that EPS54 contained a large amount of amorphous structure region, while the diffraction peak with the characteristic crystal structure was not found. Therefore, EPS54 exhibits an amorphous structure state on the whole, which is similar to the structure of polysaccharides from *Dendrobium officinale* (48).

### 3.4 Effect of EPS54 on *Helicobacter pylori* infected AGS cells

**Figure 5:**
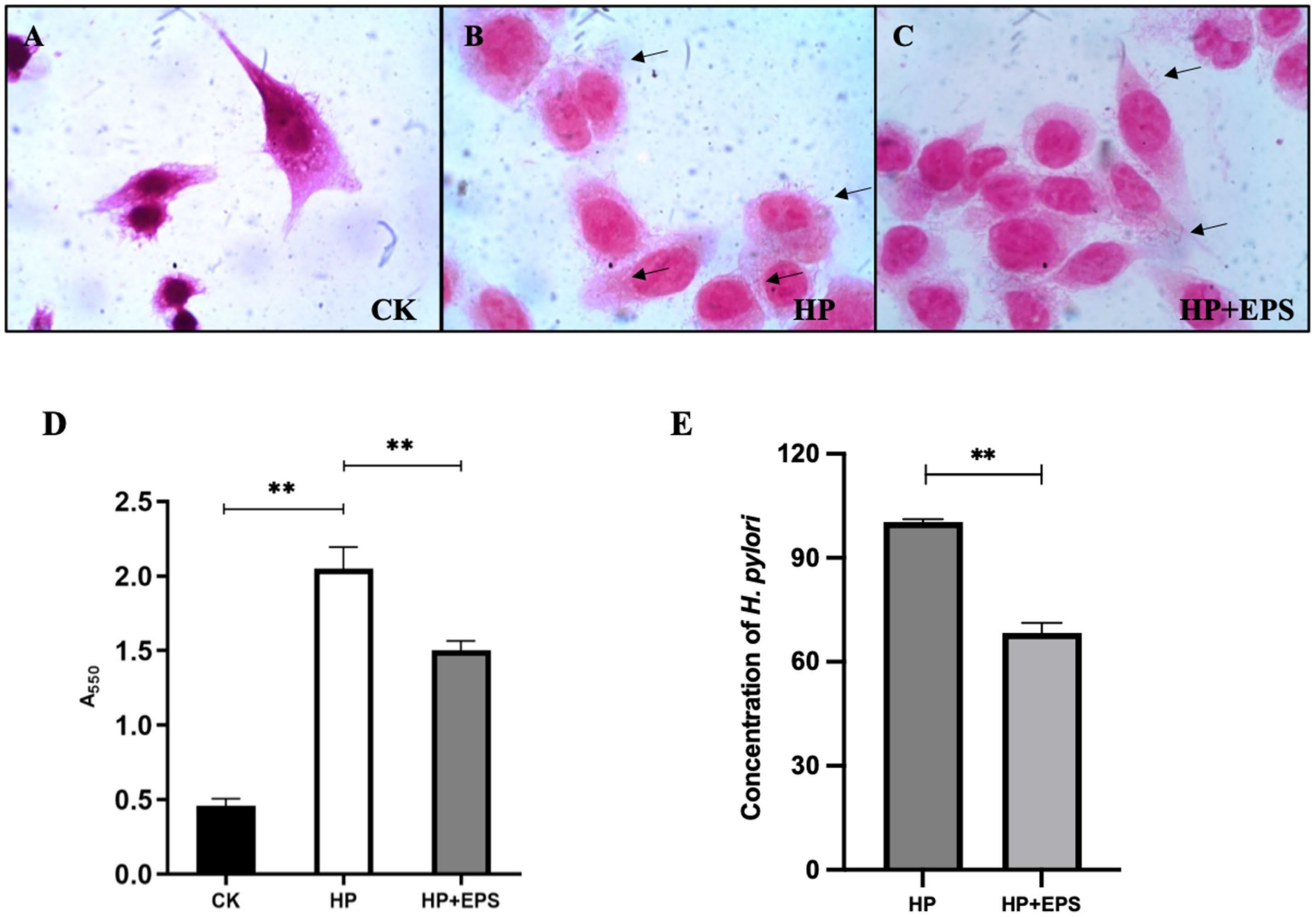
Adherence **(A–C)** (1000×), urease activity **(D)** and concentration **(E)** of *H. pylori* ZJC03 to AGS. ^**^p < 0.01.

As shown in Figure 5A, the AGS cells were polygonal in shape, and had a large cell size and a clear nuclear area, which occupied a large area of the cell. As can be seen in Figure 5B, a large amount of *H. pylori* ZJC03 were adhered to the surface of AGS cells, which were in poor state, with reduced volume and unclear edge contour, indicating that the adhesion and colonization of *H. pylori* on the cells caused a certain degree of damage to the cells. After treatment with EPS54, the cell edge became clearer, the cell morphology was improved, and the cell growth was restored to some extent (Figure 5C).

Urease activity is considered as a measure of *H. pylori* viability. As shown in Figure 5D, compared with blank group, the urease activity of AGS after infection with *H. pylori* ZJC03 (HP group) significantly increased (*p* < 0.01), treatment with EPS54 largely decreased the urease activity of AGS.

### 3.5 Effect of EPS on gastric in *Helicobacter pylori* infected mice

#### 3.5.1 Gastric tissue histopathological observation

**Figure 6:**
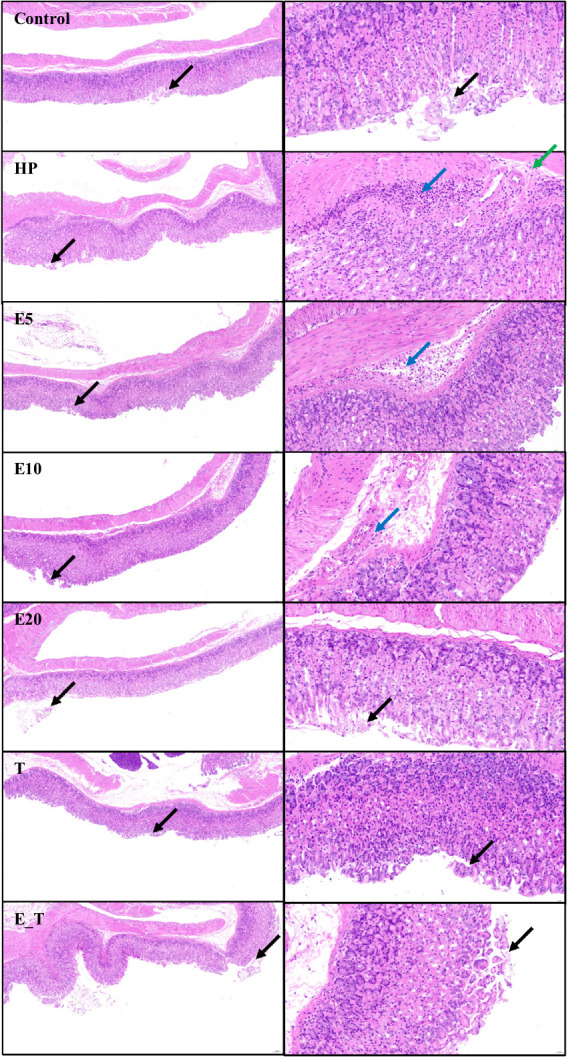
Histopathological observation of gastric tissue. Left 10×, Right 40×. Black arrow: Little epithelial cells were shed. Blue arrow: The focal infiltration of numerous lymphocytes in the submucosa. Green arrow: Submucosal edema with a small amount of lymphocyte infiltration.

The change of gastric tissue sections of the *H. pylori* infected mice after treatment with EPS54 was examined by H&E staining method. As depicted in Figure 6, in control group, the structure of the mucous membrane was clear, epithelium was complete, mucosal glands were closely arranged, and the morphology was normal, with no obvious pathological symptoms. In contrast, in the mice of HP group, little epithelial cells were shed (black arrow), the focal infiltration of numerous lymphocytes in the submucosa (blue arrow) and submucosal edema with a small amount of lymphocyte infiltration (green arrow) were observed. In the mice of T group, occasionally a small number of epithelial cells were shed (black arrow) without other obvious pathologic symptoms. In the mice treated with EPS, the number of submucosal inflammatory cells decreased significantly. In the high concentration (20 g/L) EPS treatment group, the structure of the gastric mucosa was clear, mucosal glands were closely arranged, the epithelium is relatively complete, and little epithelial cells were shed (black arrow). Although there was still a small amount of inflammatory cell infiltration in the submucosa of the EPS54 treatment group, the overall situation of inflammatory cell infiltration was alleviated with the increase of EPS dose. It can be inferred that EPS54 has the effect of alleviating gastritis reaction caused by *H. pylori* infection.

#### 3.5.2 Effects on gastric mucosal inflammatory cytokines

**Figure 7:**
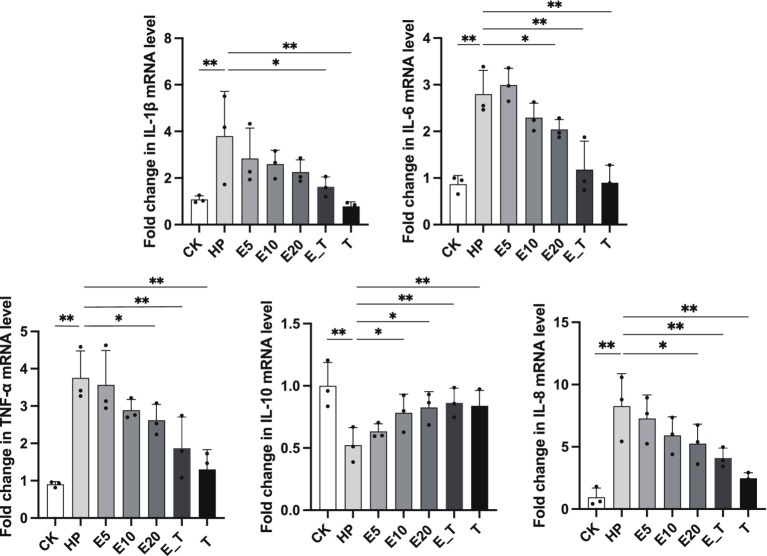
EPS54 modulates the cytokines expression level. ^*^p < 0.05, and ^**^p < 0.01.

Results of the transcription expression of five cytokines in gastric cells are presented in Figure 7. The pro-inflammatory cytokines IL-1β (*p* < 0.05), IL-6 (*p* < 0.001), IL-8 (*p* < 0.001) and TNF-α (*p* < 0.001) in the HP modeling group were significantly up-regulated compared with group CK, and anti-inflammatory cytokine IL-10 was significantly down-regulated compared with group CK (*p* < 0.01), indicating the obvious inflammation caused by *H. pylori*. In all the treatment groups, the mRNA expression levels of pro-inflammatory cytokines IL-6, IL-8, IL-1β and TNF-α in gastric cells of the *H. pylori-*induced gastritis mice were significantly reduced, while the mRNA expression of inflammatory cytokines IL-10 was significantly increased when compared to that of group HP. The effect in the three EPS treatment groups showed a dose-dependent manner. The above results agreed with those of H&E analysis, which further proves that EPS54 has obvious therapeutic effect on the inflammatory reaction caused by *H. pylori* infection.

#### 3.5.3 Gastric microbiota diversity and structure analysis

**Figure 8:**
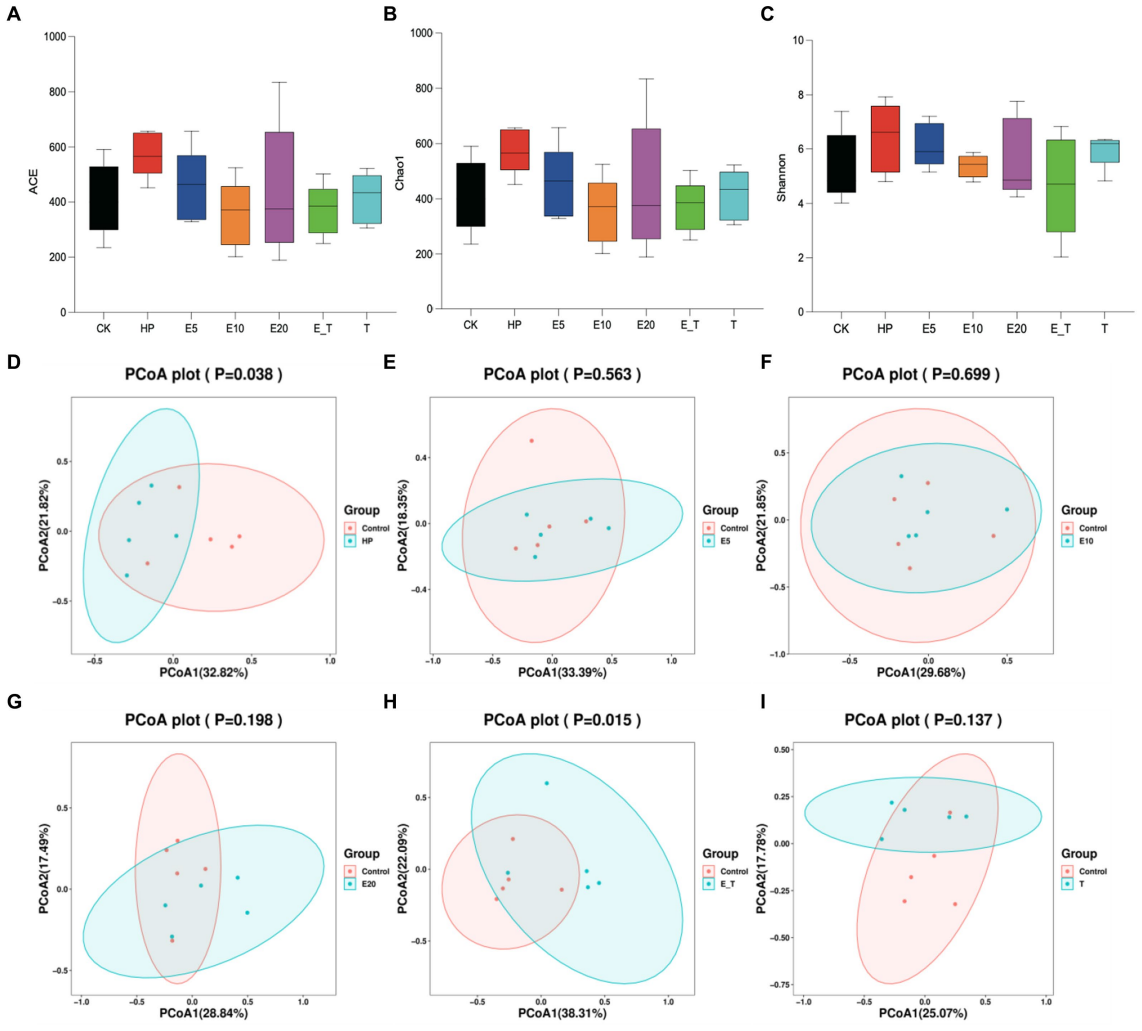
Gastric microbiota diversity analysis. **(A–C)** Alpha diversity. **(D–I)** PCoA.

In order to compare the gastric microbial composition of mice in each group, gastric contents were selected for 16S rRNA gene sequencing and classification analysis. Compared with other groups, ACE, Chao and Shannon indices were increased in group HP, indicating that the abundance and diversity of gastric microbiota changed (Figures 8A–C). Principal coordinate analysis (PCoA) analysis showed apparent clustering of gastric microbiota in different groups based on Bray–Curtis dissimilarity (Figures 8D–I). It can be seen from the results that the gastric microbiota of mice in different groups showed obvious aggregation and coincidence. The treatment with EPS54 restored the structure of gastric microbiota of mice to close to normal level.

**Figure 9:**
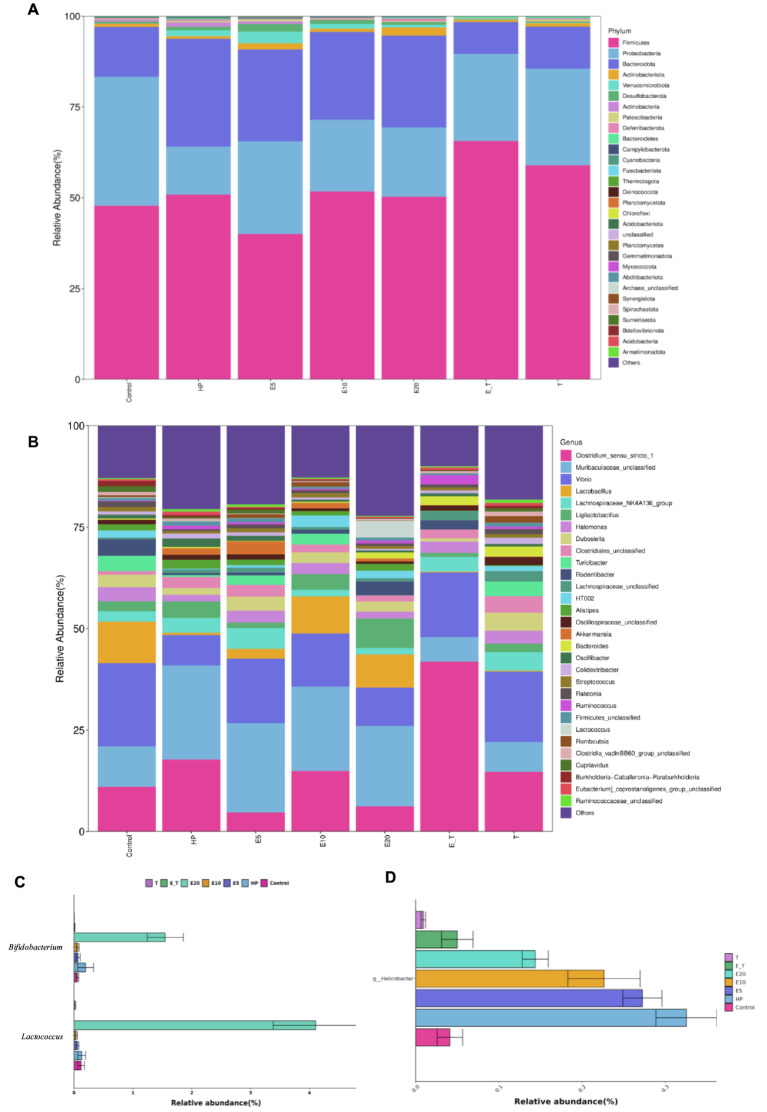
Bacterial community distribution among different groups at the phylum and genus levels. **(A)** Distribution of abundant phyla. **(B)** Distribution of abundant genera. **(C)** The relative abundance of *Lactococcus* and *Bifidobacterium*. **(D)** The relative abundance of *Helicobacter*.

The clustering results of the analysis of the gastric microbiota composition are depicted in Figure 9. At the phylum level, it was found that *Firmicutes*, *Bacteroidota*, and *Proteobacteria* were the dominant bacteria in the mice of all groups. Compared with group Control, the abundance of *Bacteroidota* and *Firmicute* increased significantly in group HP (65.33–75.91%). Triple antibiotic therapy altered the abundance of *Firmicutes* in mice, while decreased the abundance of *Bacteroidota*. Moreover, the microbiota composition of both groups T and E_T were highly similar to control group, indicating that the gastric microbiota disorder caused by *H. pylori* has been significantly improved by triple therapy, and EPS54 might act as an adjuvant to the antibiotic therapy at the phylum level.

At the genus level, microbiota in the HP group was significantly different from that in the control group, and the relative abundance of bacteria in the HP group such as *Vibrio*, *Lactobacillus*, *Lactococcus* and *Streptococcus* was significantly lower than that of the control group. It was also found that the abundance of beneficial bacteria (*Lactobacillus*, *Ligilactobacillus* and *Lactococcus*) in the EPS54-treated groups was significantly higher than that in the HP group, and the increasing trend of *Ligilactobacillus* was proportional to the concentration of EPS54. The combined statistics of *Lactobacillus*, *Lactococcus, Ligilactobacillus* and *Lactobacillaceae* HT002 showed that the abundance followed the order: E20 (21.32%) > E10 (16.29%) > Control (14.50%) > HP (4.89%) > E5 (4.45%) > T (3.59%) > E_T (1.28%).

Compared with the effect of antibiotics, high-dose EPS54 (E20) greatly increased the abundance of *Lactococcus* (4.11%) and *Bifidobacterium* (1.55%), while the abundance of these two genera in the antibiotic treatment groups (T and E_T groups) was lower than that in other groups, indicating that antibiotic treatment may have a certain inhibitory effect on the growth of beneficial bacteria such as *Lactococcus* and *Bifidobacterium*. At the same time, the abundance of *Helicobacter* on the gastric mucosa of mice in various groups was also examined. *H. pylori* has been successfully colonized in the gastric mucosa of mice by feeding *H. pylori* bacteria solution in the early stage. Antibiotic treatment significantly reduced its abundance. Also, in the EPS54 group, the abundance of *Helicobacter* decreased to varying degrees, and the effect increased with the increase of EPS54 concentration. The abundance of *Helicobacter* in E_T group decreased and was closer to that in normal mice. The combination of EPS54 and triple antibiotics was more conducive to the recovery of *Helicobacter* to normal levels. The results showed that EPS54 could promote the growth and proliferation of *Lactobacillus*, *Lactococcus* and *Bifidobacterium*, inhibit the growth of *Helicobacter*, thereby restoring the entire gastric mucosal ecology to normal levels, and gradually reducing the damage caused by *H. pylori* to the stomach.

#### 3.5.4 Linear discriminant analysis effect size analysis

**Figure 10:**
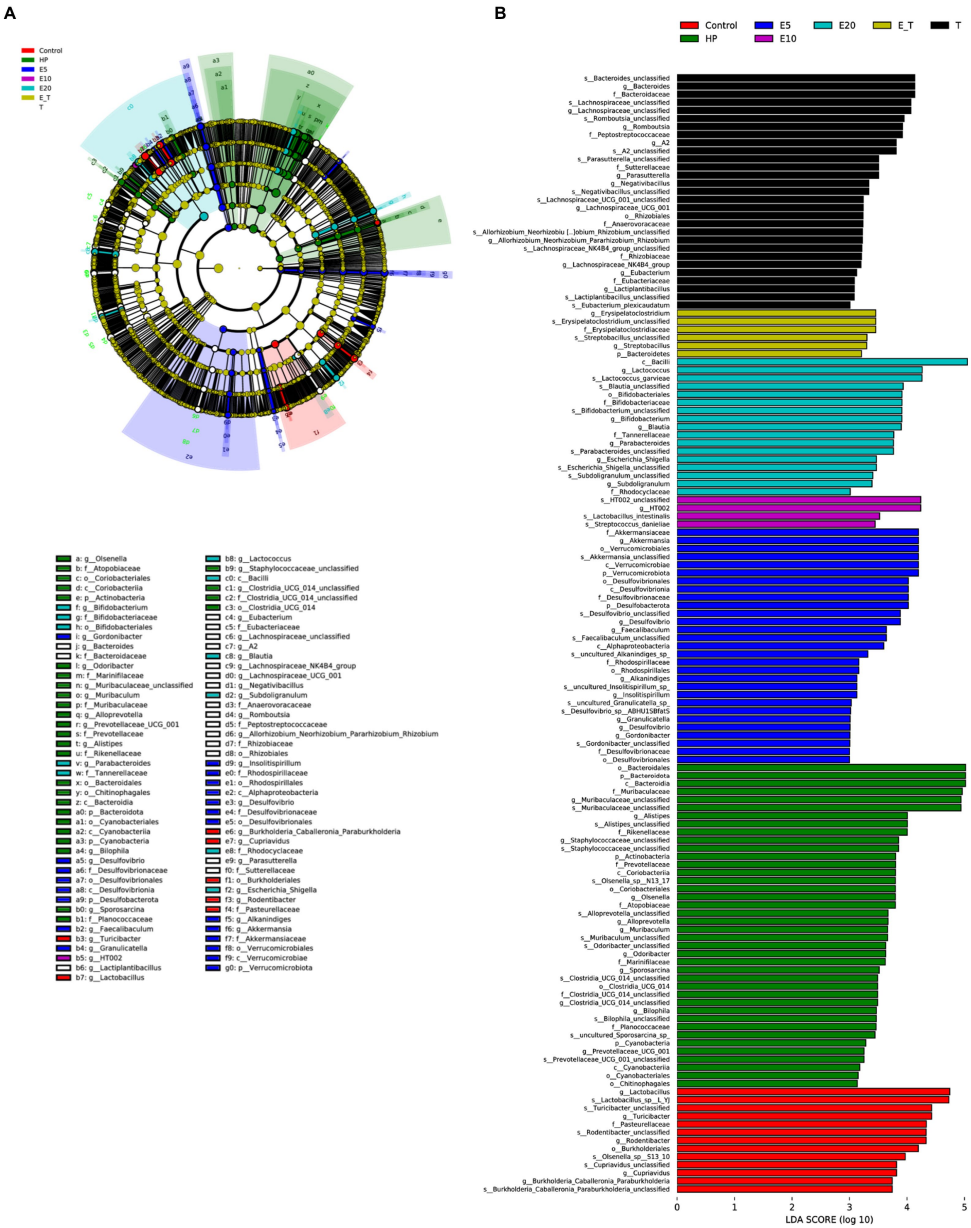
Gastric microbiota structure analysis from LEfSe. **(A)** Cladogram. **(B)** LDA.

Linear discriminant analysis effect size (LEfSe) analysis and linear discriminant analysis (LDA) were used to estimate the magnitude of the effect of each species abundance on the differential effect to identify the groups or species that had a significant differential effect on sample delineation (Figure 10). Abundant bacterial taxa observed in group HP were *Bacteroidota*, *Muribaculaceae*, *Alistipes*, *Staphylococcaceae*, *Marinifilaceae*, *Alloprevotella* and *Clostridia*. The bacterial taxa observed in the EPS54 treatment groups included *Bacilli*, *Lactococus*, *Bifidobacteriales*, *Parabacteroides*, *Streptococcus*, *Subdoligranulum* and *Blautia*, most of which belong to the beneficial bacteria for human health. The above results showed that EPS54 could improve the structure of gastric microbiota in mice infected by *H. pylori* at phylum and genus levels, and restored the composition of gastric microbiota to that in the normal mice.

## 4 Discussion

EPS produced by LABs can be divided into homo-polysaccharides (HoPS) and hetero-polysaccharides (HePS). HoPS is composed of one kind of monosaccharide, while HePS is composed of more than two kinds of monosaccharides (49). The bioactivities of polysaccharides largely depend on their structural characteristics, such as monosaccharide composition, sulfate content, glycosidic linkages, position and amounts of functional groups, molecular weight, chain conformation, and etc. (50). EPS54 developed in the present study was a HePS, mainly consisting Man, Gal, Glc, and Rib. It was reported that two polysaccharides from *Caulerpa lentillifera* containing high sulfate, mannose, galactose, xylose and glucose levels, possessed strong antibacterial activity against *H. pylori* (51). Dendrobium officinale polysaccharide (DOP) was also reported to strongly inhibit the *Helicobacter*, and DOP mainly consisted of mannose and glucose with the dominant proportions of 85.75 and 12.82% (52). Studies have found that polysaccharides with triple-helix conformation often have good biological activities and specific recognition functions, which renders them possess more potential applications (53). Therefore, the considerable anti-*H. pylori* activity of EPS54 could be associated with its complicated monosaccharide composition (especially, high level of mannose and glucose), relatively high sulfate content (13.53%), and triple-helix conformation.

*H. pylori* infection has been proved to be a high-risk factor of some diseases, including chronic gastritis, stomach cancer, peptic ulcer, functional dyspepsia and gastroesophageal reflux disease (48). Triple therapy is commonly used for the treatment of *H. pylori*. However, the problem of *H. pylori* drug resistance has been increasing. Thus, there is an urgent demand to search for safe and effective natural alternatives for prevention and treatment of the diseases induced by *H. pylori* infection. Beneficial effects of probiotics on *H. pylori-*induced inflammation have been reported in several studies. Our previous studies have shown that probiotics can alleviate the inflammation of host caused by *H. pylori*, DSS, or *Salmonella* (32, 54, 55). However, whether the secondary metabolites of probiotics contribute to this relief has not been determined, the action mechanism has also not been completely clarified. In this study, a mouse model of *H. pylori* infection with gastritis was constructed to investigate the effect of EPS on the alleviation of gastritis. EPS54 derived from *L. paracasei* ZFM54 was found to effectively ameliorate the gastric injury. It was reported that fucoidan from *Sargassum hemiphyllum* effectively inhibited *H. pylori* and reduced inflammation, thus possessing a good protection to the stomach from *H. pylori* infection (56). Agreed with previous study (56), *H. pylori* infection was also found to upregulate the expression of pro-inflammatory cytokines IL-1β, IL-6, IL-8, and TNF-α, and down-regulate the expression of anti-inflammatory cytokine IL-10. IL-6 can activate the STAT3 pathway, which is associated with gastritis and gastric cancer. Elevated expression of IL-1β can lead to a decrease in secretion of gastric acid, which is associated with development of gastric cancer. Increased production of TNF-α and IL-8 induces a strong acute inflammatory response and increases cytotoxicity. IL-10 can inhibit the activity of Th1, NK cells and macrophages, and can also induce Th2 immunity and antibody production, thus playing a critical role in gastric inflammation. Our results showed that EPS54 significantly reduced the mRNA level of IL-1β, IL-6, IL-8 and TNF-α, and promoted mRNA expression of IL-10. Therefore, it is likely that EPS54 alleviates the gastric inflammation by regulating the expression of inflammatory cytokines, including IL-1β, IL-6, IL-8, TNF-α and IL-10.

Studies have shown that *H. pylori* infection interferes with the gastric microecology, leading to the dysbiosis of gastric microbiota and inflammatory reaction (57). In the present study, the effects of EPS54 on *H. pylori*-infected cells and mice were investigated. *H. pylori* ZJC03 could adhere to and colonize AGS cells, leading to morphological changes in AGS cells. On the contrary, EPS54 could effectively reduce the colonization of *H. pylori* ZJC03 and promote the cell morphological recovery. Similarly, EPS54 significantly reduced *H. pylori* colonization in gastric mucosa of *H. pylori*-infected mice. In our previous work, *L. paracasei* ZFM54 was found to have therapeutic effect on gastritis and modulatory effect on the gastric microbiota composition in the *H. pylori-*infected mice. In the mice with preventive and therapeutic administration of *L. paracasei* ZFM54, the average relative abundance of genera *Helicobacter*, *Muribaculum*, *Staphylococcus*, *Lachnospiraceae_NK4A136_group*, *Prevotellaceae_UCG-001*, *Alloprevotella*, and *Oscillibacter* were significantly reduced when compared to the infected mice (32). In this work, the effect of EPS54 on the composition of gastric microbiota was examined at phylum and genus levels. Results showed that *H. pylori* infection significantly changed the gastric microbiota composition, and overall, the EPS54 treatment could restore the microbiota structure to that in normal group at all these taxonomic levels. Especially, it was noted that *H. pylori* infection caused a significant decrease in abundance of *Lactobacillus* and *Lactococcus* compared with group CK, while antibiotic treatment could not change this situation, indicating that antibiotic treatment may not restore gastric microbiota. *Lactobacillus* and *Lactococcus* have antibacterial activity against gastrointestinal and urinary tract pathogens (58), and can promote the release of IL-10 through TLR2 signaling (59). Thus, the increased abundance of *Lactococcus* and *Bifidobacterium* in EPS54-treated groups could be highly related to the alleviating effect of EPS54 on the gastritis induced by *H. pylori* infection. In addition, EPS was reported to possess anti-*H. pylori* activity via disrupting the cell membranes and inhibiting the colonization in gastric mucosa (60, 61). Thus, it is reckoned that EPS54 might have a certain inhibitory effect on *H. pylori*, which also contributes to alleviation of gastritis and regulation of gastric microecology. Further exploration on the action mechanism of probiotics and their EPS in controlling the *H. pylori* infection is required.

## 5 Conclusion

In the present study, *Lacticaseibacillus paracasei* ZFM54 with good EPS-producing ability was screened, and the fermentation conditions of this bacterial strain for EPS production were optimized. EPS54 produced by *L. paracasei* ZFM54 effectively reduced the colonization of *H. pylori* ZJC03 to AGS cells and promoted the recovery of cell morphology. EPS54 also significantly alleviated the symptoms of gastritis in the *H. pylori*-infected mice by down-regulating the mRNA expression levels of pro-inflammatory cytokines IL-6, IL-8, IL-1β and TNF-α and up-regulating the mRNA expression of inflammatory cytokines IL-10 in gastric cells. EPS54 was able to regulate the structure of gastric microbiota at phylum and genus levels; especially, the structure of gastric microbiota in high dosage of EPS54 treated gastritis mice was found to restore to that of normal mice. This work also provides a basis for the application of *L. paracasei* ZFM54 strain and EPS produced by this bacterial strain in food and pharmaceutical industries. In particular, the EPS54 might find applications as a food additive in fermented dairy products (such as yogurt and cheese) and ingredient of functional foods.

## Data availability statement

The original contributions presented in the study are included in the article/Supplementary material, further inquiries can be directed to the corresponding authors.

## Ethics statement

Ethical approval was not required for the studies on humans in accordance with the local legislation and institutional requirements because only commercially available established cell lines were used. The animal experiments were conducted in accordance with the Guidelines for Care and Use of Laboratory Animals and approved by the Animal Ethics Committee of Shanghai Public Health Clinical Center (Shanghai, China) with the protocol number 2021- A044-01.

## Author contributions

JY: Investigation, Methodology, Writing – original draft. ZC: Formal analysis, Investigation, Writing – original draft. QZ: Investigation, Project administration, Writing – original draft. PL: Formal analysis, Resources, Visualization, Writing – review & editing. SW: Funding acquisition, Investigation, Writing – review & editing. TZ: Supervision, Writing – review & editing. QG: Funding acquisition, Supervision, Writing – review & editing.

## Footnotes

^1^ https://www.omicstudio.cn/analysis
