# Supplementary figures and images for: Exopolysaccharide from Lacticaseibacillus paracasei alleviates gastritis in Helicobacter pylori-infected mice by regulating gastric microbiota
Source: Front Nutr. 2024 Jun 24;11:1426358. doi: 10.3389/fnut.2024.1426358 (PMC11228268; doi:10.3389/fnut.2024.1426358)

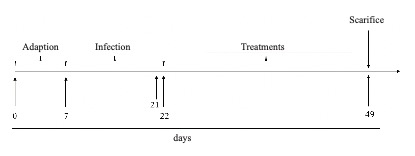

Supplement: Supplementary file 1 [file Image_1.jpg]

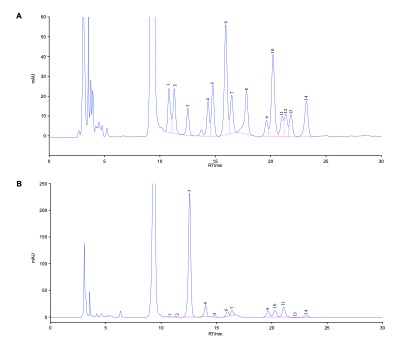

Supplement: Supplementary file 2 [file Image_2.jpg]

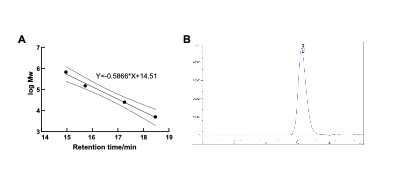

Supplement: Supplementary file 3 [file Image_3.jpg]
