# Supplementary material for: Exopolysaccharide from Lacticaseibacillus paracasei alleviates gastritis in Helicobacter pylori-infected mice by regulating gastric microbiota
Source: Front Nutr. 2024 Jun 24;11:1426358. doi: 10.3389/fnut.2024.1426358 (PMC11228268; doi:10.3389/fnut.2024.1426358)
Supplement: Supplementary file 4 [file Presentation_1.pdf]

Supplementary Materials

Exopolysaccharide from *Lacticaseibacillus paracasei* alleviates gastritis in *Helicobacter pylori*-infected mice by regulating gastric microbiota

Jianxing Yu[1,2#], Ziqi Chen[1#], Qingqing Zhou[1], Ping Li[1], Shiying Wu[1], Tao Zhou[1]*, Qing Gu[1]*

[1] *Zhejiang Key Laboratory of Food Microbiology and Nutritional Health, College of Food Science and Biotechnology, Zhejiang Gongshang University, Hangzhou, China*

[2] *College of Biological, Chemical Science and Engineering, Jiaxing University, Jiaxing, China*

Jianxing Yu and Ziqi Chen should be considered joint first authors.

*Corresponding authors.

Tel: (+86) 571 28008903. E-mail address: guqing2002@hotmail.com (Q. Gu), taozhou@zjgsu.edu.cn (T. Zhou).

ORCID: 0000-0003-4510-3639 (Tao Zhou), 0000-0001-5394-6615 (Qing Gu)

Contents

Fig. S1. Experimental design (n = 10)……………………………………….........S2

Fig. S2. I Monosaccharide composition analysis of EPS54 by HPLC..……….…..S3

Fig. S3. Mw distribution of EPS54…………………………………….……..….S4

Fig. S1.

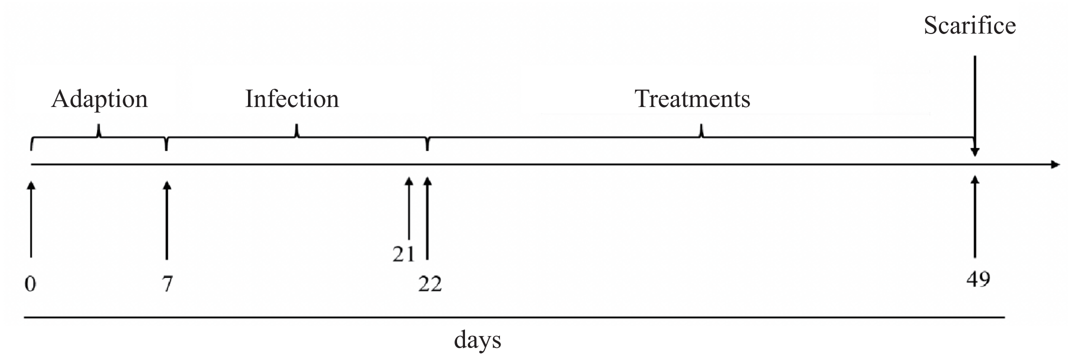

Fig. S1. Experimental design (n = 10).

Fig. S2.

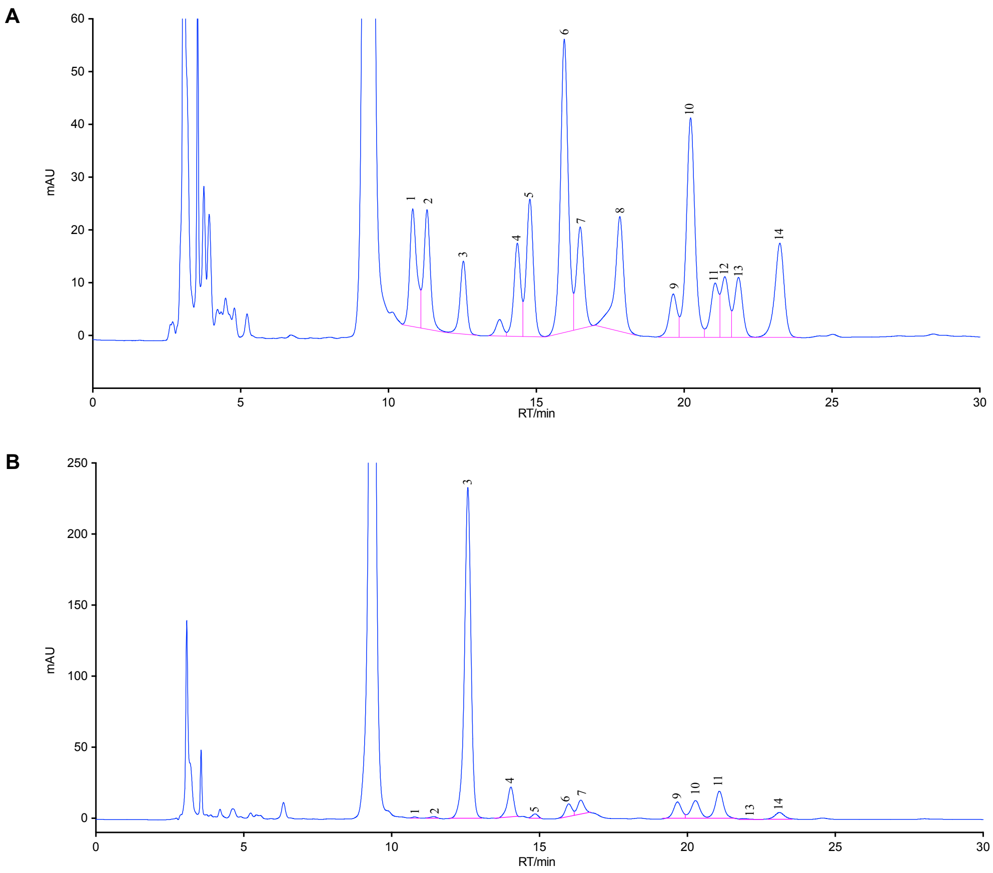

Fig. S2. Monosaccharide composition analysis of EPS54 by HPLC. 1: guluronic acid, 2: mannuronic acid, 3: mannose, 4: ribose, 5: rhamnose, 6: glucosamine, 7: glucuronic acid, 8: galacturonic acid, 9: glucose, 10: galactosamine, 11: galactose, 12: xylose, 13: arabinose, 14: L-fucose. (A: Standards; B: EPS sample).

.

Fig. S3.

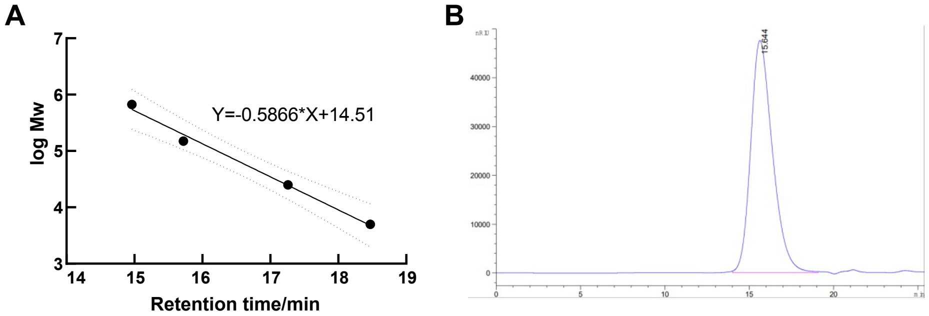

Fig. S3. Mw distribution of EPS54 (A: Standard curve; B: EPS54).
